# Human Acellular Amniotic Matrix with Previously Seeded Umbilical Cord Mesenchymal Stem Cells Restores Endometrial Function in a Rat Model of Injury

**DOI:** 10.1155/2021/5573594

**Published:** 2021-09-03

**Authors:** Shan Wang, Cheng Shi, Xiaohui Cai, Yanbin Wang, Xi Chen, Hongjing Han, Huan Shen

**Affiliations:** ^1^Peking University People's Hospital, Reproductive Medicine Center, Beijing 100044, China; ^2^Beijing Friendship Hospital, Capital Medical University, Department of Obstetrics and Gynecology, Beijing 100050, China

## Abstract

**Background:**

Abnormal endometrial repair after injury results in the formation of intrauterine adhesions (IUA) and a thin endometrium, which are key causes for implantation failure and infertility. Stem cell transplantation offers a potential alternative for some cases of severe Asherman's syndrome that cannot be treated with surgery or hormonal therapy. Umbilical cord-derived mesenchymal stem cells (UCMSCs) have been reported to repair the damaged endometrium. However, there is no report on the effects of UCMSCs previously seeded on human acellular amniotic matrix (AAM) on endometrial injury.

**Methods:**

Absolute ethanol was injected into rat uteri to damage the endometrium. UCMSCs previously seeded on AAM were surgically transplanted. Using a variety of methods, the treatment response was assessed by endometrial thickness, endometrial biomarker expression, endometrial receptivity, cell proliferation, and inflammatory factors.

**Results:**

Endometrial thickness was markedly improved after UCMSC-AAM transplantation. The expression of endometrial biomarkers, namely, vimentin, cytokeratin, and integrin *β*3, in treated rats increased compared with untreated rats. In the UCMSC-AAM group, the VEGF expression decreased, whereas that of MMP9 increased compared with the injury group. Moreover, in the AAM group, the MMP9 expression increased. The expression of proinflammatory factors (IL-2, TNF*α*, and IFN-*γ*) in the UCMSC-AAM group decreased compared with the untreated group, whereas the expression of anti-inflammatory factors (IL-4, IL-10) increased significantly.

**Conclusions:**

UCMSC transplantation using AAM as the carrier can be applied to treat endometrial injury in rats. The successful preparation of lyophilized AAM provides the possibility of secondary infectious disease screening and amniotic matrix quality detection, followed by retrospective analysis. The UCMSC-AAM complex may promote the better application of UCMSCs on the treatment of injured endometrium.

## 1. Introduction

Endometrial injury, which affects endometrial thickness and involves IUA formation, is a cause of amenorrhea, implantation failure, and infertility [1], which have been on the rise due to the continuous increase in the rates of abortions and hysterectomies in China [2, 3]. Presently, IUA formation is treated with estrogen-based intrauterine devices (IUDs) to promote endometrial growth and balloons to prevent IUA recurrence. However, IUDs can adversely affect endometrial repair as well as cause inflammation and infertility [4]. To increase the thickness of the endometrium, sildenafil and granulocyte colony stimulating factor (G-CSF) are applied [5]. However, these entities have very limited effects on the restoration of endometrial function, especially in cases of severe basal layer damage [6, 7].

With advancements in tissue engineering, mesenchymal stem cells (MSCs) have been used to repair and regenerate tissues [8]. The basal layer, which lies beneath the epithelial layer, supplies stem and progenitor cells for the production of new functional layers in each menstrual cycle [9, 10]. These cells were reported to express mesenchymal-like stem cell markers and show mesodermal differentiation characteristics [11–13]. Unfortunately, iatrogenic curettage results in the destruction of the stem cell niche, and hence, in the loss of stem cells and in the failure of endometrial regeneration. Investigators have used donor-derived bone marrow mesenchymal stem cells (BMMSCs) to repair endometrial damage, essentially triggering the expression of endometrial cell markers, increasing endometrial thickness, increasing blood vessel formation, and improving endometrial receptivity [14–17]. Other studies have demonstrated that tail vein injection of UCMSCs increased the expression of HOXA10, a biomarker of endometrial receptivity, and improved endometrial function in mice [18–20].

Studies have shown that transplanation yields different effects [21, 22]. An ideal transplantation method should meet four conditions as follows: (i) a relatively high concentration of MSCs should accumulate in the injured area, (ii) MSCs should be evenly distributed within the endometrium, (iii) MSCs should effectively adhere to the endometrium and not be squeezed out of the uterine cavity, and (iv) there should be no biological toxicity and immunogenicity. To meet these requirements, MSCs should be seeded onto a scaffold, which then be transplanted.

Human amniotic membrane (AM) is a biofilm derived from the inner layer of the human placenta. It has been used in tissue repair for over a century and has many advantages such as its similarity with the three germ layers compared with synthetic materials [23]. The pores on AM can allow water and some small molecules to pass through; although, it is difficult for inflammatory cells to infiltrate [24, 25]. AM expresses IL-1 receptor antagonist (inhibits the expression of proinflammatory factors), IL-10 (inhibits the secretion of inflammatory factors), IL-4, IL-6, IL-8, and protease inhibitors [26]. In addition, AM secretes a variety of growth factors and cytokines, including platelet-derived growth factor AA (PDGF-AA), transforming growth factor *β*1 (TGF*β*1), vascular endothelial growth factor (VEGF), and fibroblast growth factor 2 (FGF-2). It was also reported that AM can promote the proliferation of endothelial cells and the expression of growth factors and facilitate angiogenesis [27]. Therapeutic effect of Foley balloon with or without AM on IUA was compared, and menstrual recovery, readhesion, and the pregnancy rate in the AM+Foley balloon group were better than Foley balloon alone, suggesting that the AM acts as a biologically active isolator and is valid on the treatment of endometrial adhesions [28].

Despite the advantages of FAM (fresh AM), FDAAM (freeze-dried acellular amniotic membrane) should be used instead of FAM to decrease the risk of infectious diseases. AAM refers to the biological material composed of the basal layer and dense layer of AM [29]. It has characteristics of nonimmunogenicity, a 3D fiber-like structure, good biocompatibility, and natural degradation [30, 31]. Decellularization and lyophilization will decrease the levels of cytokines and other bioactive factors, while culturing UCMSCs on AAM can make up for this deficiency. If FDAAM is used as carrier, adhesions can be prevented under the premise of ensuring safety. At the same time, it provides a 3D structure for the attachment, growth, and migration of UCMSCs in vivo, furnishing a high density of these grafting cells in per unit area. In addition, certain cytokines secreted by UCMSCs can promote cell proliferation and regulate inflammation, which can promote repair of endometrial damage. Therefore, the present study constructed the UCMSC-AAM complex and detected its ability to regulate inflammation response and restore injured endometrium. The safety of AAM as cell carrier was also confirmed. There has been no study reported about MSC using AAM as carrier to cure endometrial injury diseases yet. Our results may provide insights on the innovative application of AAM as cell carrier and biofilm in tissue damage diseases and cell replacement therapy.

## 2. Materials and Methods

### 2.1. Ethics

All surgical procedures were performed on 6–8-week-old female Sprague Dawley rats (SPF level, Beijing Vital-River Laboratory Animal Technology Company, China) weighing 220–260 g. Four rats were housed per cage at a room temperature of 23–25°C, a relative humidity of 40–60%, and a light/dark cycle of 12 h/12 h. All rats had free access to food and water. All animal procedures were approved by the Laboratory Animal Ethics Committee of Peking University People's Hospital and adhered to National of Institutes of Health guidelines. All researchers involved in animal experiments were licensed by the Beijing Association on Laboratory Animal Care. The acquisition of amniotic membranes from human placentas was approved by the Ethical Review Board Committee of Peking University People's Hospital with prior written informed consent.

### 2.2. Acquisition of Human Placentas

Mothers who tested negative for the human immunodeficiency virus, syphilis, hepatitis B virus, and hepatitis C virus were registered. Human placentas were acquired from three mothers who underwent cesarean sections with no complications, and amniotic membranes were placed into containers containing saline under sterile conditions with no remaining villi.

### 2.3. Preparation of AAM

This experiment was performed in 10 cm dishes (Corning, USA) in a biological safety cabinet. AM was treated with 100 U/mL of penicillin and 100 *μ*g/mL of streptomycin (Thermo Fisher Scientific, USA) for 30 min and rinsed with sterile saline. Thereafter, it was treated with 0.1% Triton X-100 (Sigma-Aldrich) and incubated in a shaking incubator at 37°C for 36 h. AM was rinsed with saline and treated with 2.5% trypsin-EDTA (Thermo Fisher Scientific) at 37°C for 4 h. A cell scraper was used to separate the epithelial cell layer and the spongy layer. Tissues were cut into 1 cm × 2 cm fragments. A portion of the FAAM was cryopreserved in 0.1% glycerol and stored at -20°C.

### 2.4. Cell Culture and Reagents

UCMSCs were obtained from the Beijing Stem Cell Bank, which is certified by National Institutes for Food and Drug Control (Report No: SH201401380), and the Karyotype, specific surface antigens, and differentiation results were showed in Figure S1. Cells were established from umbilical cord obtained from a healthy puerpera who underwent a cesarean section. Cells were cultured in 6 cm culture dishes in *α*-MEM medium (Thermo Fisher Scientific) containing 10% fetal bovine serum (FBS) (Thermo Fisher Scientific), 100 U/mL penicillin, and 100 *μ*g/mL streptomycin. Cells were passaged with 0.25% Trypsin-EDTA and cultured at 37°C in an incubator containing 5% CO_2_/95% air. After incubation for 12 h, UCMSCs were labeled with GFP and sorted by flow cytometry. Cells were passaged for further analysis after 2–3 days. Cells from early passages (p3–p5) were used in the following experiments.

### 2.5. Preparation and Detection of the UCMSC-AAM Complex

#### 2.5.1. Preparation of the UCMSC-AAM Complex

FDAAMs with the same size (1 cm × 2 cm) were submerged in 0.5 mL of PBS in a 6 cm dish and flattened with forceps. The dish was sealed and placed in an incubator at 37°C overnight and then in a refrigerator at 4°C for 4 h, and FDAAM will firmly attached to the bottom of dish, which should be used during 1 week. Place the dish at room temperature for 1 h and aspirate the remaining liquid on the lid before use. Thereafter, UCMSCs were counted before the same number (1 × 10^6^) of cells that were seeded in each dish, shake carefully to make cells evenly distributed, and cultured at 37°C in an incubator containing 5% CO_2_/95% air, with daily changes of medium. Cells were stained with DAPI on day 3. UCMSC-AAM complex was harvested on day 5, at the accurate same time when cells were left to incubate, to maintain the consistency of cell incubation time between batches.

#### 2.5.2. Comparison of Carrying Quantity

The same number of UCMSCs was seeded on FAAM and FDAAM, followed by culturing under the same conditions. On day 3, when the cell confluency was approximately 70–80%, FAAM and FDAAM were washed three times with PBS and digested with 0.25% trypsin for 5 min. Cells were collected and counted.

#### 2.5.3. Scanning Electron Microscopy

On day 3, FAAM and FDAAM were fixed in 2.5% glutaraldehyde overnight, washed three times in PBS (15 min each time), and treated with 1% osmic acid for 2–4 h. Specimens were dehydrated in an alcohol series, dried, and immersed in isoamyl acetate overnight. Specimens were coated with gold before scanning electron microscopy (FEI, inspect s50).

### 2.6. Animal Experimental Design

#### 2.6.1. Group and Treatment

A total of 48 rats in the same phase of estrus cycle were randomly divided into four groups as follows: endometrial injury group, AMM transfer after injury group, UCMSC-AAM transfer after injury group, and normal group (*n* = 12 for each group, 6 were sacrificed at the estrus phase three cycles after surgery for histological and PCR analyses, and the others were mated for fertility tests at four cycles after surgery).

#### 2.6.2. Wright and Giemsa Stain Assay

Totally, 60 rats were selected to assess the changes of various types of cells in vaginal smears. Vaginal smears were performed on at 9 : 00 a.m. for 15 consecutive days using lavage method. 10-20 *μ*L of lavage fluid was spread evenly on the glass slide and wait for dry naturally. Wright and Giemsa staining solution (G1020, Solarbio, China) was used following the instruction.

#### 2.6.3. Establishment of the Rat Endometrial Injury Model

All surgical procedures were performed under sterile conditions. All rats (*n* = 36), except those of the normal group, were anesthetized with isoflurane, and body temperatures were maintained at 37 ± 0.5°C. The abdominal wall was opened, and the uterus was revealed. Thereafter, 0.3 mL of absolute ethanol was injected with a 1 mL insulin syringe into one side of uterus. The ethanol was aspirated five minutes later, and the uterus was rinsed with saline three times. The other side of the uterus was treated identically. The abdominal cavity was washed three times before suturing.

#### 2.6.4. Surgical Transfer of UCMSC-AAM

For the AAM/UCMSC-AAM group, transplantation was carried out three estrous cycles after endometrial injury. Rats with normal menstrual cyclicity were anesthetized with isoflurane. A 2 cm vertical incision was made through the abdominal wall to expose the uteri. Thereafter, a 0.3 cm vertical incision was made, and the AAM/UCMSC-AAM was transplanted to cover the damaged area with ophthalmic forceps.

#### 2.6.5. Fertility Tests

The uterine function was assessed by the ability to retain embryos for pregnancy. At four cycles after transplantation surgery, rats were mated at 1 : 1 ratio with sexually mature male rats, and the vaginal plugs were checked next morning. Pregnancy outcome data including pregnancy rate, number of neonatal rats, and birth weight were recorded.

### 2.7. Hematoxylin and Eosin Staining

Hematoxylin and eosin staining were used to assess the morphology of the endometrium. Tissues were embedded in paraffin, sectioned at a thickness of 5 *μ*m, and stained. Sections were examined under an inverted phase contrast microscope (Leica, German, DMIL-PH1). Endometrial thickness was measured in cross-section of the uterus as the vertical distance between the endometrial–myometrial interface and the endometrial surface. Image Pro Plus 6.0 software was used to measure endometrial thickness. Five fields in each image were selected for counting. The endometrial gland density was evaluated counted from six randomly selected fields per section under a magnification of ×400. The thickness and the morphology of endometrium were evaluated and compared among the four groups.

### 2.8. Immunohistochemistry

The expression of keratin, vimentin, and integrin *β*3 was detected by immunohistochemistry. Slides were incubated with a rabbit anti-vimentin monoclonal antibody, a mouse anti-keratin monoclonal antibody, and a rabbit anti-integrin *β*3 monoclonal antibody, followed by use of the Polink-2 Plus Polymer HRP Detection System for Mouse Primary Antibodies and the Polink-2 Plus Polymer HRP Detection System for Rabbit Primary Antibodies. The color was developed by diaminobenzidine (DAB), and nuclei were stained with hematoxylin.

Immunostaining was evaluated by two independent pathologists blinded to the information. Specimens were scored according to the intensity of the color, which was reflected by a grayscale value within Image Pro Plus 6.0 software. Five fields in each image were selected for counting. Antibodies and dilutions used in immunostaining were showed in Table S1.

### 2.9. Quantitative Reverse-Transcription Polymerase Chain Reaction (qRT-PCR)

The expression of keratin, vimentin, integrin *β*3, IL-2, TNF*α*, IFN-*γ*, IL-4, IL-10, VEGF, MMP9, and Ki-67 was assessed. Total RNA from rat uteri was isolated using TRI Reagent (Sigma) according to the manufacturer's protocol. Complementary DNAs (cDNAs) were synthesized using Trans Script One-Step gDNA Removal and cDNA Synthesis Super Mix (TransGen Biotech, Beijing, China). Real-time quantitative PCR was performed using primers and the Universal KAPA SYBR FAST qPCR Kit (Roche, Switzerland) in a 7900 Real-Time PCR System (Applied Biosystems). Glyceraldehyde 3-phosphate dehydrogenase (GAPDH) was used as a control. All experiments were repeated at least three times. Primer sequences used in real-time quantitative PCR were showed in Table S2.

### 2.10. Safety Assay

The health of the rats was closely monitored throughout the study. Biochemical tests were performed using the HITACHI LST008 System housed in the Laboratory of Peking University People's Hospital. These tests include an assessment of liver and kidney function and measurements of electrolytes such as AST, ALT, ALP, UA, UN, Ca, and P. The heart, liver, spleen, lung, and kidney of rats of the injured group, the AAM group, the UCMSC-AAM group, and the normal group were stained with HE and examined for abnormal manifestations such as tumor formation. These blood and organ tissues were acquired three estrus cycles after transplantation surgery.

### 2.11. Statistical Analysis

Statistical analysis was performed with SPSS 20.0 software (IBM, Armonk, NY, USA). Numerical data were represented as means ± standard deviation. Normality testing (Shapiro–Wilk) was performed for all experiments. For nonparametric statistics, non-Gaussian distributed data were analyzed using the Mann–Whitney *U* test and presented as populations with median values indicated by bars. For parametric statistics, data were assumed to be Gaussian distributed and analyzed using unpaired Student's *t*-test. Data was presented as a mean value with 95% confidence interval (CI). The chi-squared test was used to examine the differences between two or three proportions. *P* values <0.05 were considered to be statistically significant.

## 3. Results

### 3.1. Human Acellular Amniotic Matrix

**Figure 1:**
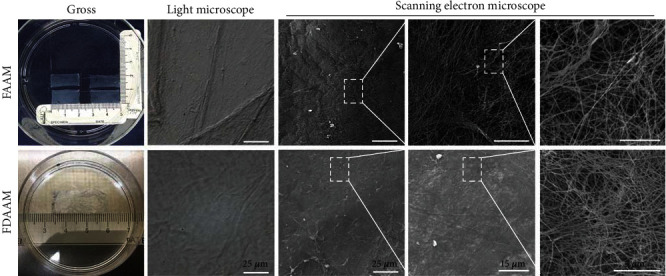
Morphology of FAAM and FDAAM. (a) Gross view, under light microscope and scanning electron microscope. Scale bars, 25 *μ*m, 15 *μ*m, and 5 *μ*m. FAAM: fresh acellular amniotic matrix; FDAAM: freeze-dried acellular amniotic matrix.

Both FAAM and FDAAM were characterized by a semitransparent and tough film. By microscopy, there were no blood vessels, nerves, and lymphatic vessels. Collagen and mesh fibers were interwoven, with the 3D network having an uneven thickness as observed by scanning electron microscopy. There were no cells on the surfaces of both FAAM and FDAAM. The FAAM mesh was relatively loose compared to that of FDAAM preserved at low temperature for half a year after lyophilization. The mesh gap on FAAM was approximately 0.5–15 *μ*m, whereas that of FDAAM was 0.2–11 *μ*m (Figure 1(a)).

### 3.2. UCMSC-AAM Carrying Capacity

**Figure 2:**
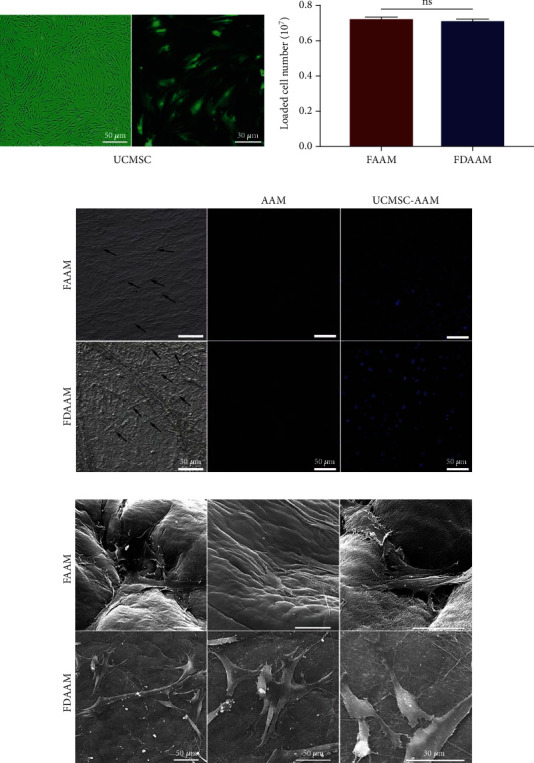
Establishment of UCMSC-AAM complex. (a) Morphology of human UCMSCs under a light microscope. Scale bars, 50 *μ*m. UCMSCs were observed by the green fluorescence of green fluorescent protein under a fluorescence microscope. Scale bars, 30 *μ*m. (b) Statistical analysis of the number of loaded cells on FAAM and FDAAM counted from six randomly selected pieces (ns: *P* > 0.05). Data were presented as mean ± standard deviation. (c) UCMSCs were detected on FAAM and FDAAM under light microscope and fluorescence microscope. Scale bars, 50 *μ*m. Arrowheads indicate seeded UCMSCs. Cell nuclei were stained with DAPI (blue). (d) UCMSC-AAM under scanning electron microscope. Scale bars, 50 *μ*m, 30 *μ*m. UCMSC: umbilical cord-derived mesenchymal stem cell; FAAM: fresh acellular amniotic matrix; FDAAM: freeze-dried acellular amniotic matrix.

UCMSCs attached to FAAM and FDAAM approximately 30 min after seeding. By day 5, UCMSCs proliferated to cover both FAAM and FDAAM with long fusiform and polygonal UCMSCs densely distributed on the surface of both matrixes as observed by fluorescent microscopy (Figure 2(a)).

By immunofluorescence staining, UCMSCs were detected on the surfaces of the matrixes before and after lyophilization (Figure 2(b)). By SEM, the morphology of UCMSCs was normal, with cells attaching to the mesh tightly (Figure 2(c)). On day 3, the average UCMSC loading was approximately 0.72 ± 0.147 × 10^7^ for FAAM and 0.71 ± 0.163 × 10^7^ for FDAAM (*P* = 0.22), and there was no significance between the groups (Figure 2(d), Table 1).

### 3.3. Assessment of Endometrial Morphology and Thickness

Three estrous cycles later after transplantation, the endometrial morphology and thickness were compared among the four groups.

**Figure 3:**
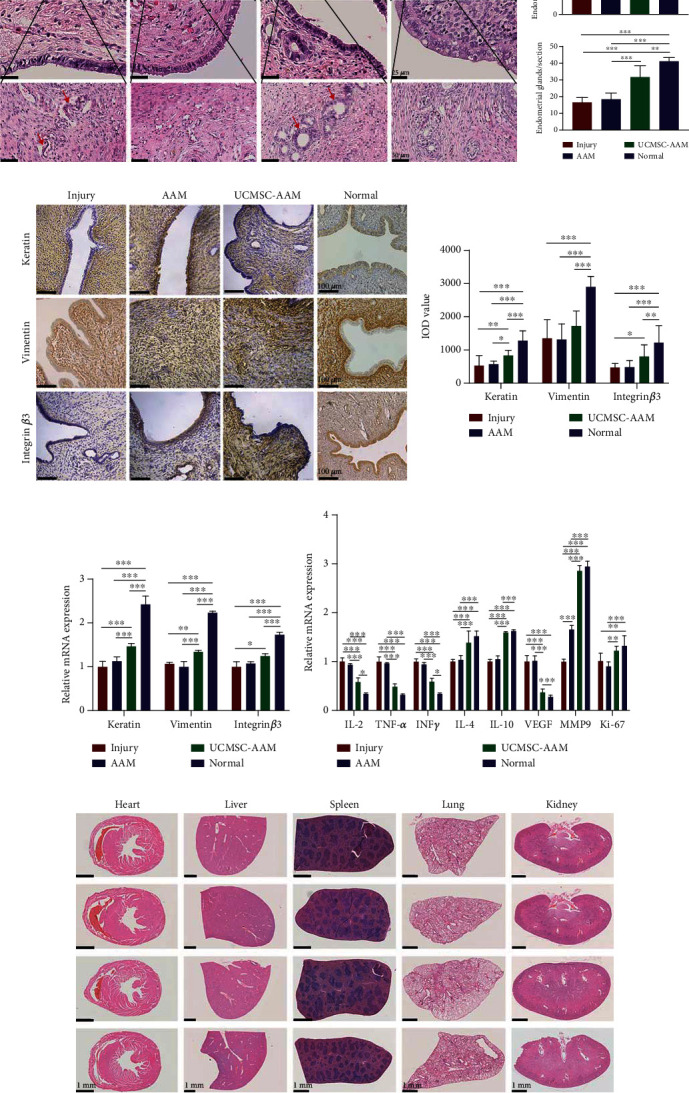
UCMSC-AAM transplantation promotes endometrial thickness, mRNA and protein expression, and regulates inflammation factors of injured endometrium. (a) Hematoxylin and eosin (H&E) staining of uterine in the injury group, the AAM group, the UCMSC-AAM group, and the normal group. Scale bars, 500 *μ*m, 25 *μ*m, and 50 *μ*m. The black arrows indicate epithelial cells, and the red arrows indicate glands. Each experiment was repeated four times. Statistical analysis of the endometrial thickness measured by Image-Pro Plus 6.0 software. ^∗^*P* < 0.05, ^∗∗^*P* < 0.01, ^∗∗∗^*P* < 0.001. (b) Immunohistochemical staining of keratin, vimentin, and integrin *β*3 for epithelial cells, stromal cells, and endometrial receptivity in endometrium of the injury group, the AAM group, the UCMSC-AAM group, and the normal group. Scale bars, 100 *μ*m. Statistical analysis of IOD (Integral optical density) value of positive areas measured by Image-Pro Plus 6.0 software. ^∗^*P* < 0.05, ^∗∗^*P* < 0.01, ^∗∗∗^*P* < 0.001. (c) Relative mRNA expressions of keratin, vimentin, and integrin *β*3 in endometrium tissues of rats in the injury group, the AAM group, the UCMSC-AAM group, and the normal group that were compared. The relative expression is the average of 2^−ΔΔCt^ ± SEM. ^∗^*P* < 0.05, ^∗∗^*P* < 0.01, ^∗∗∗^*P* < 0.001. (d) Relative mRNA expressions of IL-2, TNF*α*, IFN-*γ*, IL-4, IL-10, VEGF, MMP9, and ki67 mRNA in endometrium tissues of rats in the injury group, the AAM group, the UCMSC-AAM group, and the normal group that were compared. The relative expression is the average of 2^−ΔΔCt^ ± SEM. ^∗^*P* < 0.05, ^∗∗^*P* < 0.01, ^∗∗∗^*P* < 0.001. (e) HE staining of heart, liver, spleen, lung, and kidney in the injury group, the AAM group, the UCMSC-AAM group, and the normal group.

Endometrial thickness and morphology were significantly different between injury and UCMSC-AAM groups. For the normal endometrium, the epithelial border was intact and continuous, and the surface was smooth and wavy. Glandular epithelial and luminal epithelial cells were intact and tightly arranged, and the morphology of blood vessels and glands was normal. For the injured endometrium, the endometrium was partially interrupted and less continuous, and the luminal epithelial cells (indicated by black arrows) and the glandular epithelial cells (indicated by red arrows) showed vacuolar and a low columnar or flat shape. Glands were scattered and with incomplete edge. For the UCMSC-AAM group, the structure of glands was more complete with smooth edge, and the luminal epithelial cells and glandular epithelial cells were more tightly arranged, with intact cell membrane and rich cellular content, closing to normal cells (Figure 3(a)).

The number of endometrial glands was assessed by H&E staining. There were more endometrial glands per uterine cross-section in the UCMSC-AAM group (31.67 ± 6.24) compared with the injury group (16.67 ± 2.56, *P* < 0.001) and the AAM group (18.50 ± 3.30, *P* < 0.001), but less than the normal group (41.17 ± 1.95, *P* = 0.0048) (Figure 3(a)).

The endometrial thickness of endometrium in the UCMSC-AAM group (335.00 ± 80.07) was significantly higher than that of the injury group (335.00 ± 80.07, *P* = 0.0004) and the AAM group (367.50 ± 78.75, *P* = 0.0066), but less than the normal group (687.64 ± 98.15, *P* = 0.0005). The endometrial thickness in the AAM group increased compared to the injury group, but there was no statistically significant difference between the groups (*P* = 0.86) (Figure 3(a), Table 2).

### 3.4. Expression of Keratin, Vimentin, and Endometrial Receptivity-Related Marker Integrin *β*3

In the endometrial tissue, keratin mainly expressed in the cytoplasm of glandular epithelial or luminal epithelial cells, and vimentin expressed in the cytoplasm of stromal cells, while integrin *β*3 mainly expressed in the luminal and glandular epithelial cells during the implantation window. Under a light microscope, keratin and vimentin appear as brown particles in the cell cytoplasm, while integrin *β*3 shows brown granular particles in the cell cytoplasm and cell membrane. The positive and negative controls were showed in Figure S2.

The protein expression of keratin and integrin *β*3 in the UCMSC-AAM group was significantly higher than that in the injury group. The IOD values of keratin and integrin *β*3 in the UCMSC-AAM group were significantly higher than those in the injury group (*P* ≤ 0.001, *P* ≤ 0.01, respectively). It is worth noting that the IOD value of keratin in the UCMSC-AAM group was significantly higher than the AAM group (*P* ≤ 0.05). The IOD value of vimentin in the UCMSC-AAM group was higher than the injury group, but there was no significant difference (*P* > 0.05) (Figure 3(b), Table 3).

The mRNA expression of keratin in the UCMSC-AAM group increased compared with injury (*P* ≤ 0.0001) and AAM groups (*P* ≤ 0.001). The mRNA expression of vimentin in the UCMSC-AAM group was higher than the injury group (*P* ≤ 0.001) and the AAM group (*P* ≤ 0.001). The mRNA expression of integrin *β*3 in the UCMSC-AAM group was increased compared with the injury group (*P* ≤ 0.05), and there was no significant difference between the AAM group and UCMSC-AAM group (*P* > 0.05) (Figure 3(c)).

### 3.5. mRNA Expression of VEGF, MMP9, and Ki-67

VEGF (vascular endothelial growth factor) was reported to promote the increase of vascular permeability, degeneration of extracellular matrix, vascular endothelial cell migration, proliferation, and angiogenesis [32]. Matrix metalloproteinase 9 (MMP9) is a matrix metalloproteinase associated with extracellular matrix degradation. Cell proliferation-associated nuclear antigen Ki-67 indicates that cell proliferation was induced.

The mRNA expression of MMP9 in the UCMSC-AAM group increased compared with injury (*P* ≤ 0.0001) and AAM groups (*P* ≤ 0.0001), whereas the expression of MMP9 in the AAM group was increased compared with the injury group (*P* ≤ 0.0001). Similarly to MMP9, the expression of Ki-67 in the UCMSC-AAM group was higher than the injury group (*P* ≤0.05) and the AAM group (*P* ≤ 0.001). By contrast, the mRNA expression of VEGF in the UCMSC-AAM group was decreased compared with the injury group (*P* ≤ 0.0001) and AAM group (*P* ≤ 0.0001) (Figure 3(d)).

### 3.6. UCMSC-AAM Regulates the Expression of Inflammatory Factors In Vivo

The damage, repair, and regeneration of the endometrium are accompanied by inflammation. The mRNA expression of proinflammatory factors (IL-2, TNF*α*, and IFN-*γ*) in the UCMSC-AAM group was significantly decreased compared with the injury group (*P* ≤ 0.0001/*P* ≤ 0.0001/*P* ≤ 0.0001), whereas the expression of anti-inflammatory factors (IL-4 and IL-10) was increased (*P* ≤ 0.0001/*P* ≤ 0.0001/*P* ≤ 0.0001) (Figure 3(d)).

### 3.7. Fertility Tests

At 4 estrus cycles after transplantation surgery, rats were mated at 1 : 1 ratio with sexually mature male rats. In the injury group, AAM group, and the UCMSC-AAM group, no rats got pregnant. In the normal group, the average fetal number was 5.5 ± 0.23 per side of uterus (6, 7, 6, 5, 6, 5, 6, 6, 5, 5, 5, 4).

### 3.8. Safety of the UCMSC-AAM Transplantation In Vivo

As showed in Figure 3(e), the morphology of the heart, liver, spleen, lung, and kidney was normal, and there was no malignant tumor formation. Biochemical tests revealed no significant differences in any parameter between the UCMSC-AAM group and the normal group (*P* > 0.05) (Table 4).

## 4. Discussion

For IVF patients, a damaged endometrium is one of the reasons of repeated implantation failure, as the endometrium must be thick enough for successful embryo implantation [33]. Invasive intrauterine surgery is the most common cause of a thin endometrium and IUA formation. The basal layer is damaged, which is accompanied by the severe loss of resident stem cells and the destruction of the microenvironment [34, 35].

A variety of methods have been used to treat endometrial injury with little success. MSCs are major histocompatibility complex (MHC) class II negative and express both an immunoprivileged and immunomodulatory phenotype [36], making them a potential cell source for cell replacement therapies. The most studied among them are bone marrow stem cells (BMSCs), and UCMSCs showed a greater proliferative capacity and with no contact inhibited cell growth phenomenon when compared with BMSCs. Additionally, UCMSCs share common Wnt signaling pathways and exhibited the equal capacity to transduce canonical Wnt signaling in response to Wnt3a, a canonical Wnt with BMSCs [37]. There is no previous basic research reported that MSC xenotransplantation causes immune rejection. In a report of UCMSC subarachnoid transplantation for the treatment of spinal cord injury, 14.1% of patients had transient low fever and 1% suffered from dizziness, which were considered a stress response and disappeared after observation or conservative treatment, and no evidence-based reasons demonstrated that the mild adverse events are related to allogeneic rejection caused by MSC transplantation [38]. UCMSCs have been reported successfully used in several cases of tissue injury and inflammation [39–42]. Dai et al. transplanted UCMSCs previously seeded on synthetic collagen scaffolds into rats and observed that the labeled UCMSCs mainly distributed in the stromal layer of the damaged uterus. In addition, the number of labeled UCMSCs in the treated group was significantly higher than that in the untreated group [43]. In another study, Hu et al. transplanted artificial collagen scaffolds loaded with UCMSCs into the uteri of infertile patients with IUA formation. By 30 months, 10 out of 26 patients achieved pregnancy, with eight delivering healthy babies, one undergoing an early spontaneous abortion and one undergoing a late abortion [41]. A previous study reported that the immigration of UCMSCs into damaged tissue can be induced by the microenvironment [44]. UCMSCs can also secrete numerous cell growth factors to regulate cell differentiation and to repair damaged tissues. By contrast, UCMSCs have also been reported to inhibit the proliferation and the activation of immune cells through direct contact and secretion of regulatory factors, thereby suppressing the immune response and reducing the inflammatory damage [40, 45, 46].

An appropriate transplantation route should be selected for MSCs. Studies on the transplantation of BMMSCs and UCMSCs for spinal cord injury, myocardial ischemia, liver failure, and diabetes all showed significant differences in various aspects of cell behavior such as cell migration [41, 47, 48]. As a result, the degrees of tissue repair and functional recovery are different, which may be related to cell number, survival time, cell activity, and the microenvironment [21, 49]. Previous studies have reported that MSCs can be transplanted by intrauterine infusion, as well as by intravenous injection and direct intrauterine transplantation [42, 44, 49, 50]. From the perspective of clinical application, because of the contraction of uterus, intrauterine infusion leads to cells easily flowing out of the uterine cavity. Considering the needs of gestating a fetus, the safety of intravenous injection is controversial. Direct intrauterine transplantation of MSC-carrying graft can form a local environment with high concentration of cells, no additional migration time required, which is conducive to give full play to cells' functions. Commonly used cell scaffold materials include animal-derived type I collagen, alginate extracted from seaweed, chitosan extracted from crab shells, synthetic organic polymer materials, and inorganic materials such as calcium phosphate cement and hydroxyapatite. These cell carrier and materials have disadvantages such as high economically consuming, insufficient local stem cell concentrations, uneven distribution, easy outflow from the uterine cavity, contamination by animal-origin components, and potential toxic effects. Also, patients with endometrial injury are always accompanied by intrauterine adhesions. It is important to select a material that can serve as cell carrier as well as prevent readhesions.

AM was proved effective on the recovery of menstruation, prevention of re-adhesion, and improvement of endometrial thickness in patients with injured endometrium [28, 51].AM contains a variety of growth factors, which are conducive to the differentiation and proliferation of epithelial cells. Inflammatory factors can inhibit stromal cell proliferation and neovascularization induced by cytokines, thereby suppressing inflammation, preventing new blood vessel and scar formation to reduce adhesion recurrence, and promote tissue repair and healing. AAM is the biofilm containing the basal layer and dense layer of AM; comparing with other synthetic materials, AAM is easily acquired and economically feasible, also with no toxicity, no blood vessels, and no HLA antigens. The basal layer contains I, III, IV, V, and VIII type collagens, fibronectin, laminin, and proteoglycan. The dense layer is made up of mesh fibers, and it can prevent inflammatory cells from infiltrating the lesion. The fibers provide a scaffold for cell growth and migration, and they can enhance the attachment when UCMSCs were seeded [29, 52].What is more, AAM can effectively attach to lesions so UCMSCs proliferate and spread uniformly on the endometrial surface. AAM completely degrades 4 months after transplantation under chicken skin [25]. However, there is no report on the degradation time of AAM in the uterus.

Considering the safety of AAM, and to exclude the window period infection, FDAAM was prepared and stored at -20°C for at least 1 year after lyophilization. The donors' second blood detection was performed 3 months after the specimen was acquired, and the results were negative. FDAAM maintained normal fibrous network structure and secretion of growth factors, cytokines, and other substances. Russo et al. analyzed the levels of EGF, HGF, KGF, FGF, and TGF-*β* in fresh and freeze-dried amniotic membranes, and the levels in freeze-dried amniotic membranes were 94.43% ± 8.83, 63.78% ± 1.27, 78.55% ± 3.98, 71.64% ± 1.62, and 58.77% ± 5.07 those of fresh amniotic membranes. The levels of PDGF-AA, PDGF-BB, TIMP-1, TIMP-2, FGF2, and IL-10 were almost similar between the two types of amniotic membranes [53–55]. Decellularization of AM removed epithelial cells and avoided the possibility of immune rejection. Lyophilization can mostly preserve the active substances contained in the source materials and provides opportunities for secondary screening of infectious diseases to ensure the safety, making FDAAM a promising cell carrier and bioactive material for promotion and application. FDAAM loaded with various therapeutic cells, such as UCMSCs, combining advantages of both, has great potential in cell replacement therapy and regenerative medicine fields. At the present study, AAM served as a scaffold that maintains the cell growth and viability, as well as a biological material to separate the adhered endometrium, resulting in wound repair and IUA recurrence decline.

Our results demonstrated that UCMSCs attached to AAM and subsequently proliferated. UCMSCs showed a normal fusiform or polygonal shape, the number of cells loaded on FDAAM was identical to that of FAAM, and both matrixes have similar characteristics. In this study, no inflammatory response, infection, malignant tumors, and blood biochemical abnormalities were observed in rats receiving AAM/UCMSC-AAM transplantation.

After transplantation of UCMSC-AAM, the thickness of injured endometrium increased significantly, with morphology improvements. The mRNA and protein expression of keratin and integrin *β*3 significantly increased, reflecting the growth of endometrial epithelial cells and promotion of endometrial receptivity. The vimentin expression can reflect the growth of endometrial stromal cells, and the mRNA expression of vimentin was significantly increased; although, the IOD value was not significant on protein level. In fact, from DNA to mRNA to the protein expression, there are many factors involved in three levels of regulation including transcription, translation, and posttranslation. It cannot be ruled out that the negative regulatory factors that control the translation of vimentin are activated. Antibody specificity may also be one of the influencing factors. The expression of Ki-67 increased, indicating regeneration of the injured endometrium.

Endometrial injury is always accompanied by IUA in patients, the essence of which is fibrosis [56]. Vascular defects can lead to hypoxia, which is a potent stimulator of the VEGF expression. VEGF actions include not only mitogenic activation of endothelial cells but also increased endothelial chemotaxis, and induction of serine proteases (uPA, tPA) and collagenases triggers angiogenesis and cytoplasmic calcium accumulation, leading to endometrial fibrosis and collagen deposition. It also has a high affinity for fibrinogen and fibrin monomer, and the deposition of fibrin monomer can induce the aggregation of fibroblasts, which ultimately results in increased synthesis of the extracellular matrix (ECM). Moreover, Chaudhary and his group demonstrated that inhibition of the signaling pathways modulated by VEGF significantly attenuated fibrosis-associated diseases [57].

In consistence with the previous studies [58, 59], our results demonstrated that the mRNA expression of VEGF in endometrium in the UCMSC-AAM group decreased compared with the injury group and AAM group, higher than that of the normal group, indicating the mitigation of fibrosis after transplantation of UCMSC-AAM. It is reported that the expression of VEGF can be regulated by various growth factors and cytokines (EGF, TGF*β*, PGE2, IL-1, IL-6) [60].

Studies have reported the increase of VEGF after G-CSF perfusion and MSC transplantation, which is contrary to our results. Considering the treatment methods differ, diverse pathways are activated resulting in the different expression trends. It was reported that the recovery of injured endometrium includes three stages, the early repair stage, scar stage, and postrepair stage, and the hardness of the repair layer changes from soft to hard and then from hard to soft [61]. So, the start time to treat the injured endometrium and the time of sampling after the treatment affect the restore effect and detection results. In addition, the time point of gene and protein change is not synchronized, which can explain the inconsistent results in the VEGF expression. Still, further experiments are needed.

MMP9 is an antifibrotic factor due to its ability to degrade and remodel the ECM. It can affect ECM components, such as gelatin, elastin, and types IV and V collagen and degrade the formed fibrous tissue [62, 63]. For the UCMSC-AAM group, efficient collagen degradation in uterine scars was observed, consistent with a previous study [64]. The MMP9 expression also significantly increased in the AAM group compared with the injury group, indicating that AAM can facilitate the degradation of fibrous connective tissue and reduce fibrosis [65].

Embryo implantation is a complex process of crosstalk between various signaling molecules at the mother–fetal interface. A gradient of chemokines and cytokines is produced by endometrial cells to guide the blastocyst to the implantation site. Studies revealed that proinflammatory cytokines, such as IL-6, leukemia inhibitory factor, IL-8, and TNF*α*, participate in endometrial remodeling and immune cell recruitment into the decidua [66]. Similarly, increased IL-4 secretion from peripheral blood mononuclear cells 4 weeks after embryo transfer was reported [67]. Abnormal inflammation respond affects embryo implantation and pregnancy maintenance. After damage of the endometrium and transplantation of UCMSC-AAM, numerous proinflammatory and anti-inflammatory cytokines were produced at the injured site. The expression of proinflammatory factors, such as IFN-*γ*, TNF*α*, and IL-2, in the injury group was higher than that in the UCMSC-AAM group. Compared with the injury group, the IL-4 and IL-10 expression was noticeably enhanced in the uterus of the UCMSC-AAM group, indicating that AAM can regulate inflammation and reduce inflammation together with UCMSCs in vivo.

Still, there are limitations in the present study. There are no offspring obtained in the AAM and UCMSC-AAM group, which may relate to the time duration from transplantation to mating. Different mating time should be set to explore the appropriate repair time for endometrium, which will be carried out in our subsequent research. The badly damaged endometrium may also lead to the failure implantation of embryos, which should be modified. Also, multiple operations and possible damage of ovary are negative factors for conception and pregnancy to rats.

There is no report on the transplantation of UCMSCs using AAM as the cell carrier to treat endometrial injury, which remains a challenge in assisted reproductive technology. The good biocompatibility, toughness, and ability to regulate inflammation make AAM a promising cell carrier. Moreover, the successful preparation of FDAAM and the construction of UCMSC-FDAAM are of great significance for future clinical applications. The present report is the first study to explore the safety and effects of AAM as a cell carrier in the transplantation of UCMSCs to repair endometrial damage on a rat model.

## 5. Conclusion

Transplanted UCMSC-AAM can promote the proliferation of endometrial epithelial and stromal cells. FDAAM made it possible to assess its safety and production for future clinical applications. Thus, the UCMSC-AAM complex is beneficial in the treatment of women with endometrial injury, and AAM may be promising as a cell carrier.

## Figures and Tables

**Table 1:** Cell number loaded on the amniotic matrix before and after lyophilization ($\bar{X}\pm S$).

Group	Number of samples	Cell number (10^7^)
Fresh amniotic matrix (FAAM)	6	0.72 ± 0.147
Freeze-dried amniotic matrix (FDAAM)	6	0.71 ± 0.163
*P* value	*P* > 0.05	

**Table 2:** Endometrial thickness in the groups ($\bar{X}\pm S$).

Group	Number of samples	Thickness (*μ*m)	*P* value
Injury	12	335.00 ± 80.07	—
AAM	12	367.50 ± 78.75	*P* = 0.86
UCMSC-AAM	12	513.33 ± 148.59	*P* = 0.0004
Normal	12	687.64 ± 98.15	*P* < 0.001

*P* value: groups compared with the injury group.

**Table 3:** IOD value of endometrial cell-specific proteins and endometrial receptivity protein in the groups ($\bar{X}\pm S$).

Group	Keratin	Vimentin	Integrin *β*3
Injury	535.02 ± 325.89	1363.33 ± 598.59	481.25 ± 131.39
AAM	580.75 ± 99.09	1325.50 ± 502.93	492.92 ± 212.88
UCMSC-AAM	841.67 ± 165.87	1728.58 ± 484.90	815.58 ± 373.83
Normal	1288.07 ± 290.45	2902.36 ± 313.99	1229.79 ± 504.23

**Table 4:** Blood chemistry results three estrus cycles after UCMSC-AAM transfer.

	Normal	AAM-MSC	*P* value
AST (U/L)	128.1	117.8	*P* > 0.05
ALT (U/L)	47.1	50.6	*P* > 0.05
ALP (U/L)	138.8	130.7	*P* > 0.05
GGT (U/L)	1.4	0.9	*P* > 0.05
TP (g/L)	53.05	60.45	*P* > 0.05
ALB (g/L)	36.7	40.4	*P* > 0.05
A/G	2	2	—
UN (*μ*mol/L)	7.46	7.24	*P* > 0.05
CRE (*μ*mol/L)	57	48	*P* > 0.05
UA (*μ*mol/L)	145.6	131.1	*P* > 0.05
LDH (U/L)	525	496	*P* > 0.05
CK (U/L)	696.3	712.9	*P* > 0.05
Ca (mmol/L)	2.585	2.542	*P* > 0.05
P (mmol/L)	2.921	2.781	*P* > 0.05
Glu (mmol/L)	7.64	8.74	*P* > 0.05
CHO (mmol/L)	1.774	1.523	*P* > 0.05

AAM: acellular amniotic matrix; *P* value: UCMSC-AAM group compared with the normal group.

## Data Availability

All data and figures used to support the findings of this study are included within the article.

## Abbreviations

AM: Amniotic membrane
AAM: Acellular amniotic membrane
FAAM: Fresh acellular amniotic membrane
FDAAM: Freeze-dried acellular amniotic membrane
UCMSC: Umbilical cord mesenchymal stem cell
IUA: Intrauterine adhesions
IUDs: Intrauterine devices
G-CSF: Granulocyte colony stimulating factor
MMP9: Matrix metallopeptidase 9
BMSCs: Bone marrow mesenchymal stem cells
MSC: Marrow mesenchymal stem cell
PDGF-AA: Platelet-derived growth factor AA
TGF*β*1: Transforming growth factor *β*
VEGF: Vascular endothelial growth factor
FGF2: Fibroblast growth factor 2
CI: Confidence interval
AST: Aspartate aminotransferase
ALT: Alanine aminotransferase
ALP: Alkaline phosphatase
UN: Urea nitrogen
IOD: Integrated optical density
SEM: Scanning electron microscope
EGF: Epidermal growth factor
PGE2: Prostaglandin E2
IFN-*γ*: Interferon-*γ*
TNF*α*: Transforming growth factor-*α*
ECM: Extracellular matrix
TIMP-1: Metallopeptidase inhibitor 1
TIMP-2: Metallopeptidase inhibitor 2.

## Supplementary Materials

Supplementary 1 Figure S1: UCMSCs have stable karyotype, express specific surface antigens, and possess multilineage differentiation potential. (a) UCMSCs maintain normal karyotype after multiple passages. (b) Flow cytometry analysis of specific surface markers in human UCMSCs. (c) Differentiation assays of human UCMSCs. Osteogenesis differentiation was confirmed by immunofluorescence staining with anti-Osteocalcin antibody. Scale bars, 50 *μ*m. Adipogenesis differentiation was confirmed by immunofluorescence staining with anti- FABP-4. Scale bars, 50 *μ*m. Chondrogenesis differentiation was confirmed by immunofluorescence staining with anti-Aggrecan antibody. Scale bars, 20 *μ*m. UCMSC: umbilical cord-derived mesenchymal stem cell. Figure S2: positive and negative controls for keratin, vimentin, and integrin*β*3 staining. (a) Negative and positive control for keratin in thymus of SD rat (Scale bars, 50 *μ*m), vimentin in testis of SD rat (Scale bars, 250 *μ*m). and integrin*β*3 in ovary of C57BL/6 N mouse (Scale bars, 50 *μ*m).

Supplementary 2 Table S1: antibodies and dilutions. Table S2: primer sequences used in real-time quantitative PCR.
