# Supplementary material for: Human Acellular Amniotic Matrix with Previously Seeded Umbilical Cord Mesenchymal Stem Cells Restores Endometrial Function in a Rat Model of Injury
Source: Mediators Inflamm. 2021 Sep 3;2021:5573594. doi: 10.1155/2021/5573594 (PMC8438588; doi:10.1155/2021/5573594)
Supplement: Supplementary 1 — Figure S1: UCMSCs have stable karyotype, express specific surface antigens, and possess multilineage differentiation potential. (a) UCMSCs maintain normal karyotype after multiple passages. (b) Flow cytometry analysis of specific surface markers in human UCMSCs. (c) Differentiation assays of human UCMSCs. Osteogenesis differentiation was confirmed by immunofluorescence staining with anti-Osteocalcin antibody. Scale bars, 50 μm. Adipogenesis differentiation was confirmed by immunofluorescence staining with anti- FABP-4. Scale bars, 50 μm. Chondrogenesis differentiation was confirmed by immunofluorescence staining with anti-Aggrecan antibody. Scale bars, 20 μm. UCMSC: umbilical cord-derived mesenchymal stem cell. Figure S2: positive and negative controls for keratin, vimentin, and integrinβ3 staining. (a) Negative and positive control for keratin in thymus of SD rat (Scale bars, 50 μm), vimentin in testis of SD rat (Scale bars, 250 μm). and integrinβ3 in ovary of C57BL/6 N mouse (Scale bars, 50 μm). [file 5573594.f1.docx]

**
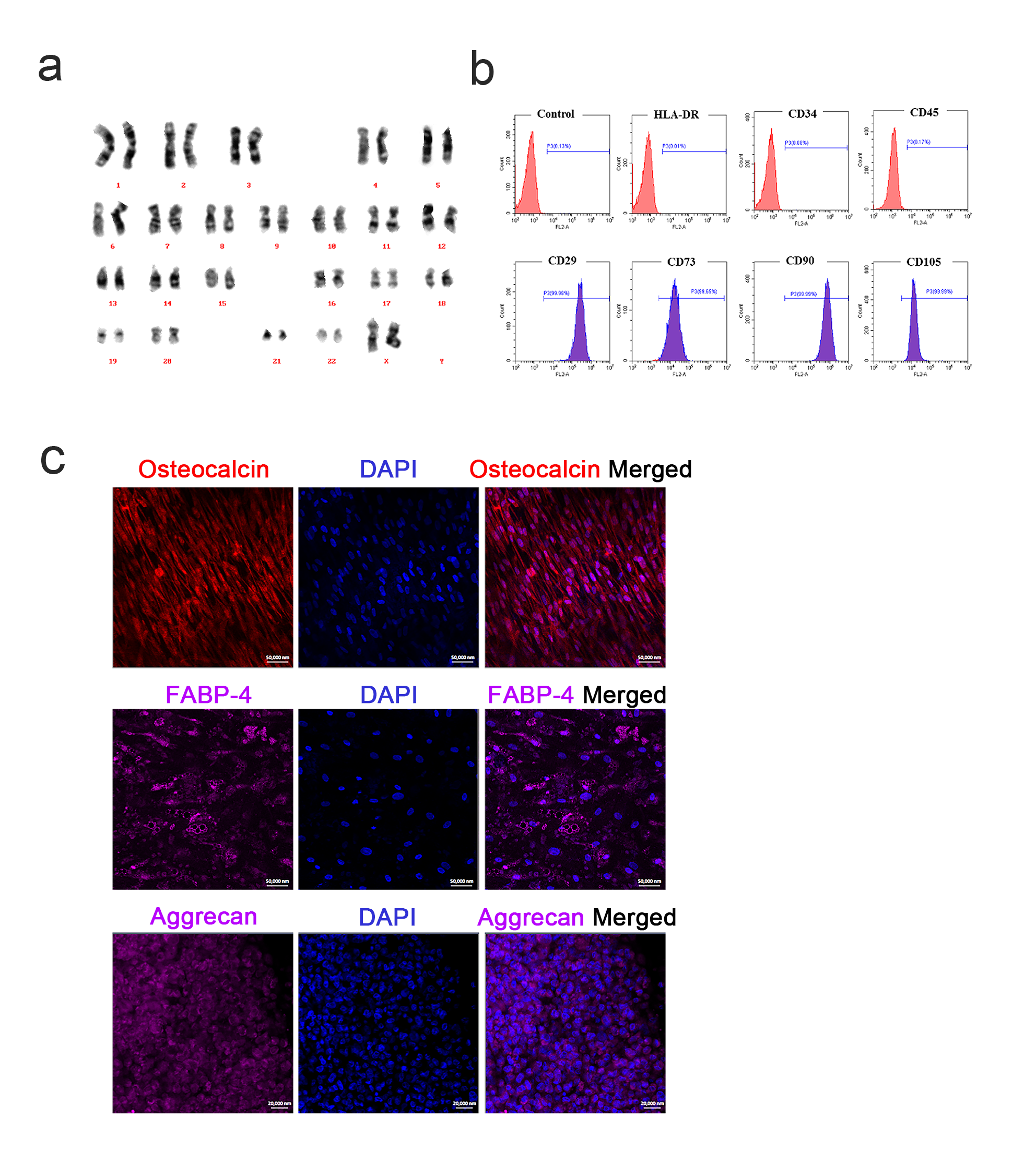
**

**Fig.S1** UCMSCs have stable karyotype, express specific surface antigens and possess multi-lineage differentiation potential.

**a** UCMSCs maintain normal karyotype after multiple passages.

**b** Flow cytometry analysis of specific surface markers in human UCMSCs.

**c** Differentiation assays of human UCMSCs. Osteogenesis differentiation was confirmed by immunofluorescence staining with anti-Osteocalcin antibody. Scale bars, 50 μm. Adipogenesis differentiation was confirmed by immunofluorescence staining with anti- FABP-4. Scale bars, 50 μm. Chondrogenesis differentiation was confirmed by immunofluorescence staining with anti-Aggrecan antibody. Scale bars, 20 μm.

UCMSC: umbilical cord-derived mesenchymal stem cell


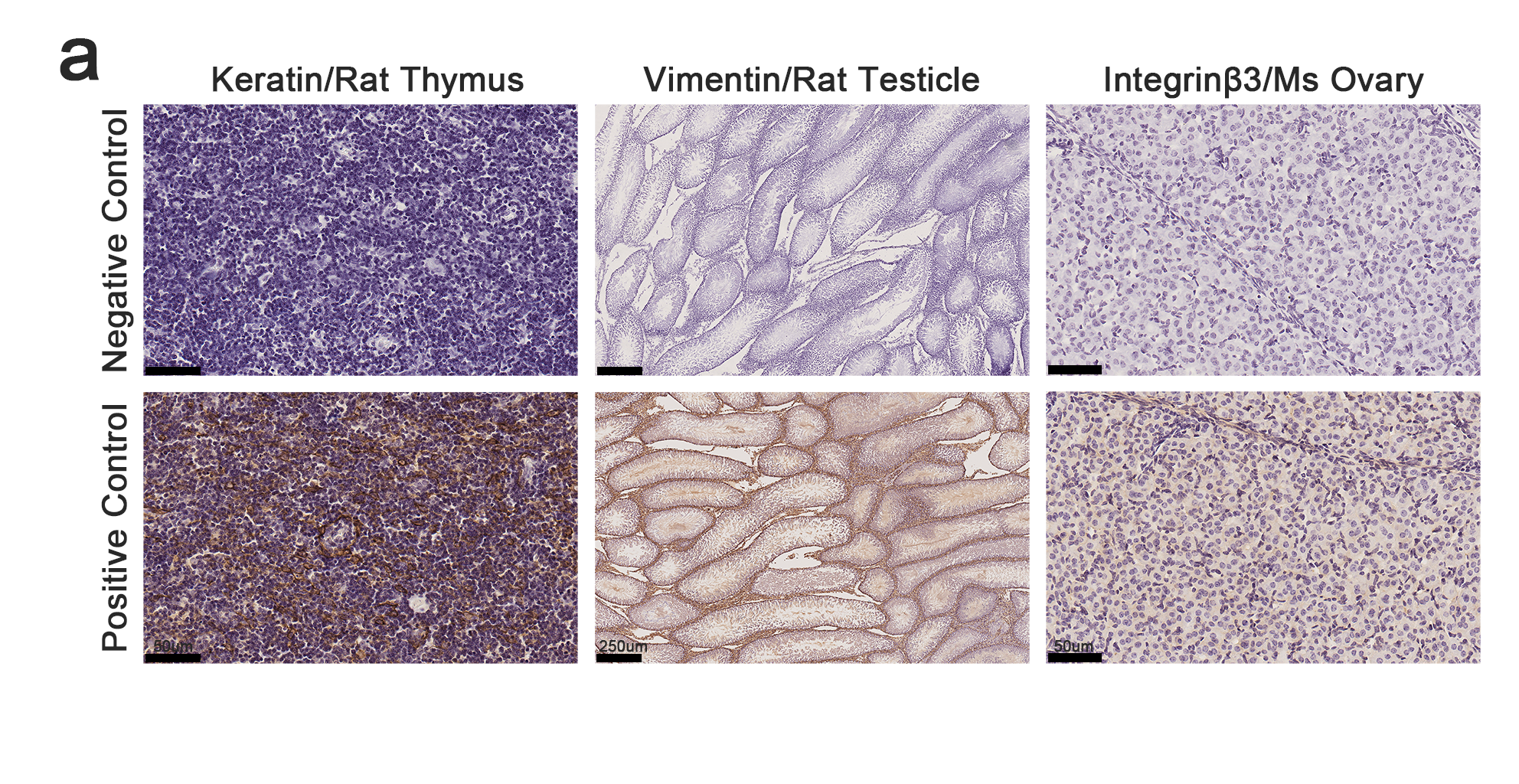


**Fig.S2** Positive and negative controls for keratin, vimentin and integrinβ3 staining.

**a** Negative and positive control for keratin in thymus of SD rat (Scale bars, 50 μm), vimentin in testis of SD rat (Scale bars, 250 μm) and integrinβ3 in ovary of C57BL/6N mouse (Scale bars, 50 μm).
