# Supplementary material for: Human Acellular Amniotic Matrix with Previously Seeded Umbilical Cord Mesenchymal Stem Cells Restores Endometrial Function in a Rat Model of Injury
Source: Mediators Inflamm. 2021 Sep 3;2021:5573594. doi: 10.1155/2021/5573594 (PMC8438588; doi:10.1155/2021/5573594)
Supplement: Supplementary 2 — Table S1: antibodies and dilutions. Table S2: primer sequences used in real-time quantitative PCR. [file 5573594.f2.doc]

Table S1. Antibodies and dilutions

| Antibody | Vendor | Cat. no. | Concentration |
| --- | --- | --- | --- |
| Keratin | Abcam | ab8068 | 1:100 |
| Vimentin | Abcam | ab92547 | 1:200 |
| Integrinβ3  Polink-2 Plus Polymer HRP Detection System for Mouse Primary Antibodies | Abcam  ZSGB-Bio | ab210515  PV-9005 | 1:100  1:100 |
| Polink-2 Plus Polymer HRP Detection System for Rabbit Primary Antibodies | ZSGB-Bio | PV-6001 | 1:100 |

Table S2. Primer sequences used in real-time quantitative PCR

| Gene name | Primer sequence |
| --- | --- |
| rat-GAPDH-qPCR-F | GCAAGGATACTGAGAGCAAGAG |
| rat-GAPDH-qPCR-R  rat-Vimentin-qPCR-F  rat-Vimentin-qPCR-R  rat-Keratin-qPCR-F  rat-Keratin-qPCR-R  rat-integrinβ3-qPCR-F  rat-integrinβ3-qPCR-R | GGATGGAATTGTGAGGGAGATG  GTCCGTGTCCTCGTCCTCCTAC  AGGTGCGGGTGGATGTGGTC  CCTATGCGGTTGGAAGTGGCTATG  CACCAATACCACCACCAAGTCCTC  TCAATGCCACCTGCCTCAACAAC  CTGAAGCTCACCGTGTCTCCAATC |
| rat-TNF-α-qPCR-F | CCCAATCTGTGTCCTTCTAACT |
| rat-TNF-α-qPCR-R | CAGCGTCTCGTGTGTTTCT |
| rat-VEGFA-qPCR-F | GAAGACACAGTGGTGGAAGAAG |
| rat-VEGFA-qPCR-R | ACAAGGTCCTCCTGAGCTATAC |
| rat-Ki-67-qPCR-F | CTGCAGAGAAGGTTGGGATAAA |
| rat-Ki-67-qPCR-R | CTGACTTTGCCCAGAGATGAA |
| rat-TGF-β1-qPCR-F | AGAGCCCTGGATACCAACTA |
| rat-TGF-β1-qPCR-R | CAACCCAGGTCCTTCCTAAAG |
| rat-MMP9-qPCR-F | GCTGCTCCAACTGCTGTATAA |
| rat-MMP9-qPCR-R | TGGTGTCCTCCGATGTAAGA |
| rat-IFN-γ-qPCR-F | GTGAACAACCCACAGATCCA |
| rat-IFN-γ-qPCR-R | GAATCAGCACCGACTCCTTT |
| rat-IL-2-qPCR-F | GCAGGCCACAGAATTGAAAC |
| rat-IL-2-qPCR-R | CCAGCGTCTTCCAAGTGAA |
| rat-IL4-qPCR-F | GGTGAACTGAGGAAACTCTGTAG |
| rat-IL4-qPCR-R | TCCAGGAAGTCTTTCAGTGTTG |
| rat-IL-10-qPCR-F | AGTGGAGCAGGTGAAGAATG |
| rat-IL-10-qPCR-R | GAGTGTCACGTAGGCTTCTATG |
